# Fostering Hotel-Employee Creativity Through Micro-Level Corporate Social Responsibility: A Social Identity Theory Perspective

**DOI:** 10.3389/fpsyg.2022.853125

**Published:** 2022-04-27

**Authors:** Naveed Ahmad, Zia Ullah, Esra AlDhaen, Heesup Han, Luis Araya-Castillo, Antonio Ariza-Montes

**Affiliations:** ^1^Faculty of Management Studies, University of Central Punjab, Lahore, Pakistan; ^2^Faculty of Management, Virtual University of Pakistan, Lahore, Pakistan; ^3^Leads Business School, Lahore Leads University, Lahore, Pakistan; ^4^Marketing Department, College of Business and Finance, Ahlia University, Manama, Bahrain; ^5^College of Hospitality and Tourism Management, Sejong University, Seoul, South Korea; ^6^Facultad de Economía y Negocios, Universidad Andrés Bello, Santiago, Chile; ^7^Social Matters Research Group, Universidad Loyola Andalucía, Córdoba, Spain

**Keywords:** corporate social responsibility, employee creativity, work engagement, extra roles, hotel, social identity theory

## Abstract

Due to globalization, a dynamic business environment, and stiff rivalry, the importance of employee creativity (EC) has increased in the current era more than ever before. The hotel sector has no exception, rather the need for creativity is high in this sector because most hotels operate in ways that are easy to imitate. Recently, researchers have paid attention to micro-level corporate social responsibility (ML-CSR) and have linked it to achieve different employee-related outcomes such as EC. However, the above relationship was less explored in a hospitality context. To bridge this gap, the current analysis aims to investigate the relationship of ML-CSR and EC with the mediating effect of work engagement (WE) in the hotel sector of a developing country. The study also attempts to extend the boundary of social identity theory in a collectivistic culture to explain the link between ML-CSR and EC. The data were collected from hotel employees (*n* = 461) and were analyzed with the help of structural equation modeling. The findings validated that ML-CSR positively influenced EC, and WE mediated this relationship. The current work offers different contributions to the theory and the field which are discussed in detail.

## Introduction

The discussion of creativity and innovation in workplaces is ever increasing in the existing literature (Aksoy, 2017; Awan et al., 2019) as it is evident from the literature that creative organizations are expected to overrun their rivals (Manfredi Latilla et al., 2018; Jeong and Shin, 2019) through their superior performance. Given this, organizations that want to be a pioneer in their field or want to maintain this position desire to develop strategies aimed at the development of service and production processes in a creative way (Sutanto, 2017). The stiff competitive situation, in every business sector, has further increased the importance of creativity for contemporary businesses (Ismail, 2015). Perhaps this is why many global players, in the current age, not only realize the importance of employee creativity (EC), but they encourage their workforce to be engaged in creative processes. Google is a relevant case as it encouraged employees to spend 20% of their working hours in creative thinking. The results of Google’s 20%-time project have been incredible because Google was able to introduce Gmail as an outcome of EC (Mccracken, 2014). Organizations without any meaningful differentiation in their market offerings are unlikely to achieve any significant success in the long run (Darvishmotevali et al., 2020). The importance of EC and innovation in organizations cannot be ignored and may be regarded as key factors to the success of an organization.

The literature argues that employees’ perceptions about an organization can significantly influence their attitude and behavior (Cropanzano et al., 2001; Hur et al., 2018; Deng et al., 2022). In this regard, corporate social responsibility (CSR) has been found a key factor to shape the attitude and behavior of employees at the workplace. A plethora of research studies have acknowledged the importance of CSR to achieve different employee-related outcomes (Khaskheli et al., 2020; Kim et al., 2020; AlSuwaidi et al., 2021). However, the link of micro-level CSR (ML-CSR) with EC in the context of hospitality sector was less explored previously. To define ML-CSR, this study uses the definition of Rupp and Mallory (2015) who referred ML-CSR as “CSR activities of an organization at the level of individuals.” Though some researchers have investigated EC in a CSR framework (Hur et al., 2018; Guo et al., 2021), however, such a sparse explanation is insufficient to advance the field. Therefore, the current research study aims to test the relationship between ML-CSR and EC with the mediating effect of work engagement (WE). The mediating role of WE was also proposed previously (Chaudhary and Akhouri, 2018, 2019; Asif et al., 2019), nevertheless, WE as a mediator in a CSR framework, especially in the context of the hospitality sector, was less focused in previous studies.

The current study considers the hotel sector of Pakistan to test the proposed relationships. This sector was intentionally selected for the current survey due to the following reasons. First, like other service sectors, the hotel industry is also an industry that is recognized as a labor-intensive industry in which employees play a crucial role in achieving different organizational objectives. Thus realizing the importance of employees in such a labor-intensive sector is critical for every hotel. The authors’ argument here is that employees are prone to show their commitment toward extra roles, such as creativity, when they are given a supportive environment. In this regard, ML-CSR engagement of an organization is one of those strategic enablers that is well observed by the employees to enhance their extra-role commitment for an organization. Other scholars also acknowledged the importance of CSR to enhance the extra-role behavior of employees (Afridi et al., 2020; Chaudhary, 2020). Thus, it will be important for this sector to see what the contribution of ML-CSR is for inducing employee creative capability. Second, globally the hotel sector is one of those service sectors in which a high turnover rate is evident (Glenn, 2016; Erica, 2019; Amanda, 2020). The same holds in the case of Pakistan’s hotel sector where high turnover is reported (Shaikh and Zahid, 2016). In this regard, the authors argue that the image of a socially responsible hotel is something that attracts the attention of the employees and urges them to stay with a hotel. Put simply, a socially responsible hotel has a positive perception among employees, and it is expected that in such hotels, the employee will stay longer and even will be willing to contribute more for its success by performing extra-roles. Different scholars have also acknowledged that a socially responsible hotel is in a better position to receive a positive evaluation from employees as compared to the ones which do not have this image (Dedeoğlu et al., 2018; Wang et al., 2018). Lastly, in the hotel sector, physical elements have a significant impact on novelty perceptions of customers. For instance, renovating the decorations or adapting to technological advancements could lead customers to have high novelty value perceptions for a specific hotel. However, these kinds of physical creative initiatives are easily imitable. Therefore, physical creative investments could result in a short-term competitive advantage. On the contrary, personnel delivering services in a hotel play a very significant role in terms of competitive advantage as the creativity through employees is not easy to imitate because such type of creativity is idiosyncratic in detail. Hence, employees through their creative skills can bring a long-term competitive advantage in this sector.

The current study has some important contributions to the extant literature. To begin with, the current study adds significantly to the hospitality sector from an ML-CSR perspective. In this regard, majority of studies have focused on the macro-level of CSR previously (Rhou et al., 2016; González-Rodríguez et al., 2019; Franco et al., 2020). Similarly, the current study adds to the existing literature of organizational behavior in a way that it introduces ML-CSR as an enabler to promote EC at the workplace. The majority of the previous studies in the domain of ML-CSR have established the link between ML-CSR and employees’ pro-environmental behavior (Afsar et al., 2018; Suganthi, 2019; Tian and Robertson, 2019). However, the link of ML-CSR with EC, especially in the hospitality sector, did not receive due attention in the current literature. Moreover, the current study introduces WE as a potential mediator between ML-CSR and EC. The concept of WE is well-linked with EC in the existing literature (Sarfraz et al., 2019; Khan et al., 2020), yet it is unclear from the previous studies how ML-CSR through WE can foster EC. Another important contribution of the current study is that it extends the boundary conditions of social identity theory (SIT) by Tajfel (1978). This theory provides a foundation to explain employees’ behavior at the workplace. Generally, in the previous studies, SIT was limited to explaining employees’ formal behaviors like job satisfaction (Brunetto and Farr-Wharton, 2002; Todd and Kent, 2009), motivation (Van Knippenberg, 2000), and performance (Gundlach et al., 2006). However, linking this theory to explain employee’s extra-role behavior (creativity) was less emphasized.

The remainder of the current article is divided into four major sections. Section “Theory and conceptualization” discusses the related literature to establish different hypotheses of the current study, followed by the methodology section in which the sampling process, data collection, and instrumentation are discussed. Sections “Results” and “Discussion,” respectively, include results and analysis section in which the statistical results are discussed and a final discussion of the results in comparison to the previous studies along with implications.

## Theory and Conceptualization

This research study uses the lens of SIT to formulate hypotheses. Having its roots in the seminal work of Tajfel (1978), this theory postulates that the self-concept of an individual, at least in parts, is derived from the perceptions of an individual about a social group he/she belongs. With this regard, Ashforth and Mael (1989) were among the first who extended SIT from social psychology to an organizational context. Indeed, SIT has largely been employed by different previous researchers to explain individual behavior in a specific context. Even, with regard to the CSR framework, researchers have considered this theory to explain individual behavior (Turker, 2009a; Abdullah et al., 2017; Ko et al., 2020; Ahmad et al., 2022). Following the crux of this theory, the authors of this draft argue that employees working in a socially responsible organization are inclined to identify themselves with an ethical organization. In this context, the CSR engagement of an organization portrays its image of a good corporate citizen, and thus employees are expected to identify themselves strongly with such organizations. The study of Hu et al. (2020) mentioned that SIT explains why there is value congruence between employees and a socially responsible organization. Indeed, when employees see that their organization acts ethically for the larger interest of society and community, it motivates them to strongly identify themselves with such an ethical organization. This strong identification of employees leads them to regard organizational success as their success, and therefore they engage themselves in attitudes and behaviors including extra-role behaviors that could lead their organization toward success. Moreover, in a socially responsible organization, employees feel secure and assume their work is more meaningful and put greater effort by taking risks, being open, and thinking in creative ways of doing things. This argument that employees strongly identify themselves with a socially responsible organization is also supported by several extant scholars (Hameed et al., 2016; Chaudhary and Akhouri, 2018; Opoku-Dakwa et al., 2018).

A work environment characterized by a sense of responsibility and an aspiration to help all stakeholders encourages the employees to think in new and creative ways to solve different societal problems (Aguinis and Glavas, 2019). A socially responsible organization is expected to equip its workforce with an environment that is safe and less restricted such that employees are ultimately urged to take the risk, explore new experiments, and think about creative ideas to better serve the stakeholders (Kim et al., 2021). According to Woodman et al. (1993), creativity can be regarded as “the creation of novel, valuable and useful ideas related to product/service, process or procedures by a person in a social system.” The study of Abdelmotaleb et al. (2018) also used the above definition of EC in a CSR framework. An organization that shows greater concern for the wellbeing of all stakeholders including the internal stakeholders (employees), inspires the employees, motivating them to support such organizations through their extra roles, and their creative behavior is one such extra-role (Abdelmotaleb et al., 2018; Tong et al., 2019). Employees working in a socially responsible organization are expected to have a high intrinsic motivation to identify themselves with such organizations (Skudiene and Auruskeviciene, 2012). This high level of intrinsic motivation of employees induces their creative capability (Ryan and Deci, 2000). Hur et al. (2018) identified that ML-CSR activities of an organization positively affect the sense-making process of an employee, which in turn is linked with their creativity. Following SIT, the positive sense-making of an employee about a socially responsible organization urges him to support his organization beyond the formal obligation by performing different extra roles (Tong et al., 2019). According to SIT, the employees are expected to perform different extra roles for their organization because they identify themselves with the organization and they have a strong desire to boost the reputation of the group to whom they belong (organization in the current case).

A plethora of research studies have argued that developing an ethical context such as CSR in an organization cultivates a job attitude among employees which is beneficial for others (Skudiene and Auruskeviciene, 2012; Lee et al., 2013; Gao and He, 2017; Chen et al., 2019). Specifically, EC is closely linked with the ethical conduct of an organization because employees’ perception of their organization’s ethical orientation fosters organizational mindfulness pertinent to creativity and innovation (Hur et al., 2018). Organizations that cultivate an environment characterized by CSR orientation help their employees develop a feeling of “unconstrained work environment” in which they are encouraged to think creatively to produce better products or services for the community and the organization (Glavas and Piderit, 2009; Jones et al., 2017). To conclude, the ML-CSR orientation of an organization builds positive sense-making for a socially responsible organization among the employees, and they feel proud to identify themselves with such an organization. Thus, in line with SIT, employees desire to strongly identify themselves with an organization that is socially responsible and put every effort to earn a good reputation for their firm. Moreover, they are expected to engage themselves in different extra roles for the success of their organization. Hence, the authors propose the following hypothesis:

**Hypothesis 1:** ML-CSR of an organization is expected to positively associate with employee creativity.

Corporate social responsibility and WE research is relatively new, but there are studies that prove that there is a mutual relationship between CSR and WE. Jia et al. (2019) found that WE depends upon employees’ positive perceptions of CSR. Gürlek and Tuna (2019) also validated a direct link between CSR and employee engagement. Nazir and Islam (2020b) suggest that the rationale for the direct link between ML-CSR and employee WE lies in greater meaning and value in work due to the CSR engagement of an organization. Explicitly, CSR allows enterprises to go beyond formal obligations that appear in their formal value statement (Kim and Stepchenkova, 2021; Srivastava and Singh, 2021) and focuses on the wellbeing of the workers beyond the formal routines. In turn, it sends signals to employees about the company’s values, which is consistently reported in a host of research that found a positive correlation between CSR and employee value congruence (Munro et al., 2018; Donia et al., 2019; Rodrigo et al., 2019).

In addition, CSR can also be a way to get more value in the work. Various researchers acknowledge CSR as a way for employees that increase their meaningfulness at the workplace as the employees assume that being the worker of a socially responsible organization they are not only working for the betterment of their organization but also for the betterment of the community (Lythreatis et al., 2020; Nazir and Islam, 2020a; Nazir et al., 2021). In addition, Grant et al. (2008) found that this sense of working for the betterment of the community makes employees feel better for themselves, which ultimately improves their organizational identification to enhance their self-concept in line with SIT.

This analysis defines WE in line with Schaufeli et al. (2002), who argued that WE is a positive, performance-enhancing state of mind with strength, commitment, and absorption in a work environment. Engaged workers are strong, enthusiastic, deeply immersed in the work, and work toward organizational goals and organizational prestige (Shuck and Wollard, 2008). Again, in line with SIT, when employees work for an organization that is value-driven, that is, a socially responsible organization, they are more likely to develop strong identification with it. This sense of belonging within the organization can increase the motivation of employees to display different extra-role attitudes and behaviors that support an organization to achieve extraordinary outcomes. One of such extra role is the employee’s engagement in creativity at the workplace. Therefore, when employees have a good understanding of their organization’s CSR activities, they are more likely to bring more energy, as well as higher commitment and absorption to the job. Therefore, it can be expected that the employees will be able to maintain their real zest at the workplace in order to maintain the reputation of their organization and contribute to its success.

The authors argue here that CSR perception of employees of their organization enhances their WE, as validated by various extant scholars (Gao et al., 2018; Hu et al., 2020; Ismael and Yesiltas, 2020). This enhanced level of WE as a result of employees’ CSR perception of their organization is expected to motivate them to initiate creative and risk-taking behaviors driven by an inner feeling to meet social and organizational challenges. In addition, engaged employees are more likely to have positive emotions toward expanding their intellectual capability, which pushes them to explore new and creative ways of doing the work (Hur et al., 2018).

These positive psychological stimuli encourage employees to learn, discover new information, and build intelligent and creative solutions for the socially responsible organization they work for Jones et al. (2017). Moreover, engaged employees retain more energy needed for creativity than non-engaged employees (Cohen-Meitar et al., 2009). Supporting the above discussion, several contemporary scholars validated a positive link between WE and creativity (Toyama and Mauno, 2017; Asif et al., 2019; Bakker et al., 2020). The above discussion and literary arguments encouraged the authors to propose the following hypotheses.

**Hypothesis 2:** Work engagement positively relates to employee creativity.

**Hypothesis 3:** Work engagement mediates the relationship between ML-CSR and employee creativity.

## Methodology

### Population, Sample, and the Data Collection

The authors targeted the hotel sector of Pakistan to test the proposed relationship of the current analysis. The hotel business has been in operation in Pakistan since 1947, providing considerable support to the Pakistani economy. At the start, this sector did not develop, but over time, the hotel industry in Pakistan grew significantly. Pakistan has registered itself as an important destination for investors in the hotel sector in recent times. This sector is seeing significant growth, which is likely to continue in the future (Pakistan Hotel Association, 2021). At present, the hotel sector in Pakistan is characterized by various international players including Avari, Marriot, Carlton, Regent, Hotel Mövenpick Karachi, Pearl Continental, Ramada Plaza, and others along with various national players. Large cities of Pakistan like Lahore, Karachi, Faisalabad, Rawalpindi, and Islamabad are famous for their industrial activities, and these businesses very often arrange their meetings and seminars in different hotels. Perhaps this is the reason that in these cities various national and international hotels have been operating for many decades (Kamal, 2019). The authors in this regard selected Lahore, Karachi, and Islamabad cities of Pakistan as the sampled cities to collect the data from different hotels. In this regard, the authors put extensive efforts to identify the hotels that were practicing different CSR activities in these three cities. The authors verified the CSR engagement of the selected hotels from their websites and through personal visits with the spokesperson of these hotels. In this regard, all upscale hotels were practicing different CSR-related practices, and some of these hotels also share their CSR achievements on the hotel webpage to be communicated with diverse stakeholders.

The unit of analysis of the current analysis is employees serving in the sampled hotels. The authors contacted the Human Resource Department of the selected hotels to seek their permission for the data collection and necessary arrangements during the data collection phase. Those who showed their willingness to facilitate the data collection process in the larger interest of the industry and academia were then approached by the authors on a specific date(s) and time(s). The authors, first of all, asked for informed consent from each respondent; for this purpose, a separate sheet was attached with each questionnaire. The author used a printed version of the questionnaire (paper-pencil technique) to collect the data for the current analysis. The participants of the survey were also given the choice to withdraw from filling out the questionnaire if they felt uncomfortable. Moreover, the authors observed the Helsinki Declaration protocols to strictly observe ethical standards.

There is a debate on the sample size in a research project. While some authors suggest larger sample size (Zikmund et al., 2013) can give a better representation of the population, others think that an unnecessary larger sample can give birth to the issue of type-II error (Sekaran and Bougie, 2016). To address the issue carefully, the authors distributed 650 questionnaires, expecting a return of less than 500 as mentioned by Sekaran and Bougie (2016), a reasonable sample size. In this regard, the authors distributed these questionnaires among different employees serving in the selected hotels. Finally, the authors were able to collect 461 completed questionnaires which means the response rate of the current analysis is almost 70%. To address the issue of social desirability, the authors took different measures. For example, the items of a variable were presented in the questionnaire randomly. This was done to break any intended sequence of answering by the respondents (Sun et al., 2020; Ahmad et al., 2021a). Similarly, the respondents were realized by the authors that it was important for a respondent to respond appropriately so that the quality of the derived results could be maintained (Ahmad et al., 2021b). Further, the respondents were also assured that the information, they provided to the authors, will be kept confidential (Kong et al., 2021).

### Instrument

Construct operationalization is an important issue in social research studies (Wong, 2013). The authors in this regard were logical to use already existing measures to operationalize the latent constructs of the current study. One advantage of using the pre-established instrument is that they have their pre-tested reliability and validity (Hyman et al., 2006). Thus, the authors used the pre-existing scale of ML-CSR from the study of Turker (2009b). This scale was composed of twelve statements. A sample statement was “This hotel encourages its employees to participate in voluntary activities.” Originally, this scale consisted of seventeen items, however, five items were related to customers and government, therefore, the authors did not include these five items. Other scholars like Murtaza et al. (2021), Shah et al. (2021), and Ahmad et al. (2022) also employed the same twelve items scale in their studies. In like manner, the statements of WE were extracted from the study of Schaufeli et al. (2006) who introduced a nine-item scale to tape the latent construct of WE. A sample item was “I am immersed in my work.” Finally, the authors drew the five items of EC from the study of Coelho and Augusto (2010), who adapted it from Scott and Bruce (1994) A sample item was “I try to be as creative as I can in my job.” The questionnaire was divided into two parts, among which the first part was related to the demographic information of the respondents, and the second part was related to the construct-related items which were rated on a five-point Likert scale. The questionnaire was also presented to the experts in the field in order to verify its appropriateness to serve the purpose of the current analysis (Adnan et al., 2021; Awan et al., 2021). Further, to maintain the ethical standards, the authors followed the general standards of the Helsinki Declaration (Alam et al., 2021; Ullah et al., 2021). The profile of the respondents is shown in below Table 1.

**TABLE 1:** Sample profile (*N* = 461).

Demography	Description	%
Gender	Male	66.4
	Female	33.6
Age	25 or below	17.3
	26–30	22.8
	31–35	29.2
	36–40	20.6
	Above 40	10.1
Academic qualification	14 years or less	33.7
	16 years	48.4
	Above	17.9
Department	Front office	13.5
	Kitchen	22.1
	Human resource	11.6
	Marketing	19.8
	Others	33.1
Experience (years)	3 or less	17.7
	4–7	41.3
	8–10	21.4
	Above	19.6
Job position	Manager	38.7
	Non-manager	61.3

## Results

### Common Method Bias

The current analysis collected data with the help of a self-reported measure at a specific point of time. Moreover, all the information of the variables was collected from the same respondent, which raises the concern for common method bias (CMB). According to Podsakoff and Organ (1986), CMB is a strong concern to be addressed by the researchers when both input and output variables are perceptually measured (as is the case in the current analysis) and derived from the same individual. CMB usually occurs when variance in responses are due to the instrument instead of the actual predispositions of the sample that an instrument aims to uncover. Put simply, the instrument is a source of bias and will produce an unusual biasness that will not be genuine. Therefore, the results generated by a biased instrument will not be liable as CMB is likely to create a false internal consistency. Hence, CMB may generate systematic measurement errors that will inflate or deflate the observed relationship and can cause either a type-I or type-II. So in order to detect whether the issue of CMB exists in the current analysis, Harman (1976) one-factor analysis was performed. To do this, all the items were loaded onto a single factor. Following the guideline of Harman, the potential issue of CMB were checked. In this regard, if the output of one-factor analysis reveals that a single factor is explaining a significance of the total variance (more than 50% variance), then it is assumed that CMB is a potential issue to be addressed. However, in the current analysis, the largest variance explained by the single factor was 29.33% which is well below the threshold level of 50%. Thus, it is assumed that the potential issue of CMB is minimal and does not require any further adjustment.

### Establishing Validity and Reliability

The authors conducted a confirmatory factor analysis (CFA) in AMOS software (Table 2). The results revealed that the item loadings were significant (λ > 0.5, and ideally λ > 0.7). These results indicated that the theoretical model of the current study fits well to the data. The results for convergent validity (CV) have also been reported in Table 2. The average variance extracted (AVE) assesses the variance caused by the construct relative to the variance caused by measurement error (Fornell and Larcker, 1981). In this regard, if the value of AVE is lower than 0.5, it means that the largest variance explained is not due to the construct but due to measurement error. As it can be seen from Table 2, all the values of AVE were significant (all AVEs > 0.5), meaning that there is no issue of CV in the current analysis (Gefen et al., 2000). In like manner, the authors also tested the composite reliability (CR) of each construct. The outputs of CR analysis for all of the three constructs were significant as each construct’s CR value was greater than 0.7, thus the criterion for CR in this analysis was well achieved (Table 2).

**TABLE 2:** Item loadings, convergent validity, and reliability results.

Item code	λ	λ^2^	Var (ε)	Σλ^2^	Items	AVE	CR
WE-1	0.80	0.64	0.36				
WE-2	0.72	0.52	0.48				
WE-3	0.74	0.55	0.45				
WE-4	0.79	0.62	0.38				
WE-5	0.83	0.69	0.31				
WE-6	0.80	0.64	0.36				
WE-7	0.71	0.50	0.50				
WE-8	0.76	0.58	0.42				
WE-9	0.75	0.56	0.44	5.303	9	0.59	0.93
EC-1	0.73	0.53	0.48				
EC-2	0.84	0.71	0.29				
EC-3	0.87	0.76	0.24				
EC-4	0.84	0.71	0.29				
EC-5	0.89	0.79	0.21	3.49	5	0.70	0.92
MLCSR-1	0.81	0.66	0.34				
MLCSR-2	0.71	0.50	0.49				
MLCSR-3	0.78	0.61	0.39				
MLCSR-4	0.74	0.55	0.45				
MLCSR-5	0.82	0.67	0.33				
MLCSR-6	0.79	0.62	0.38				
MLCSR-7	0.86	0.74	0.26				
MLCSR-8	0.77	0.59	0.41				
MLCSR-9	0.74	0.55	0.45				
MLCSR-10	0.83	0.69	0.31				
MLCSR-11	0.82	0.67	0.33				
MLCSR-12	0.87	0.76	0.24	7.611	12	0.63	0.95

*λ, Item loadings; CR, composite reliability; Σλ^2^, sum of square of item loadings; Var (ε), error variance; EC, employee creativity; and WE, work engagement.*

### Correlations

Table 3 shows the results of inter-correlations, means, standard deviations, maximum shared variance (MSV), and average shared variance (ASV). As can be seen from Table 3, that all values of correlation are positive and significant meaning that these positive correlations are providing initial support toward the validation of hypotheses in the current analysis. It is also to be noted that none of the correlation values is greater than 0.85, which means the issue of multi-collinearity is not a serious concern here (Kline, 2015). Moreover, the values of standard deviations (SD) are less than 1.00, which means the data is close to its mean. Likewise, the values of MSV and ASV are less than the values of AVEs for each construct, which validates that CV and discriminant validity (DV) are well-established (Hair et al., 2010). The values of Cronbach alpha (α) ranged from 0.93 to 0.77, which is indicative of the internal consistency of each construct (Nunnally, 1994).

**TABLE 3:** Correlation, discriminant validity, and model fit indices.

Construct	ML-CSR	WE	EC
ML-CSR	**(0.77)**	0.34**	0.38**
WE		**(0.93)**	0.29**
EC			**(0.89)**
Mean	3.87	4.13	3.98
SD	0.58	0.63	0.55
MSV	0.14	0.15	0.14
ASV	0.13	0.11	0.11

SD, standard deviation; **, significant values of correlation; bold diagonal, Cronbach alpha; MSV, maximum shared variance; ASV, average shared variance; EC, employee creativity; and WE, work engagement.

Finally, the results of measurement model confirmed the good fit between theory and data (χ^2^ = 4708.482, *df* = 983, *p* < 0.01 χ^2^/*df* = 4.789, RMSEA = 0.057, CFI = 0.929, NFI = 0.906). The value of χ^2^/*df* is fewer than 5, which indicates there is a good fit between theory and the data (Wheaton et al., 1977). Likewise, as per the recommendation of Byrne (2013), CFI and NFI values were also significant (both values are greater than 0.90). Lastly, the value of RMSEA is less than 0.060, which is also significant.

### Hypotheses Testing

Different hypotheses of the current analysis were tested by using the structural equation modeling technique (SEM) through AMOS software. In this context, the authors were in line with several researchers (Matthews, 2017; Richter et al., 2020; Thakkar, 2020), who advocated strongly in the favor of SEM to deal with complex models involving moderator(s), mediator(s) or both. SEM is a second-generation co-variance-based data analysis technique that contains more advanced and sophisticated techniques, in contrast to conventional regression analysis through SPSS. To validate the hypotheses, SEM analysis was performed in two steps. The first step was the direct effect model and the second step was the mediated model. The first step was carried out without involving any mediator in the structural model. The results of the direct model have been shown in Table 4. As per the finding of the direct effect model, there was a significant effect of ML-CSR on WE and EC (β1 = 0.31, β2 = 0.38, *p* < 0.05) as indicated by their respective beta values and *p-*values. Thus, based upon the statistical pieces of evidence, it is established that both hypotheses (H1 and H2) of the current analysis are found to be significant, and H1 and H2 are therefore accepted. Moreover, the model fit values were also significant (χ^2^ = 3814.281, *df* = 847, *p* < 0.01 χ^2^/*df* = 4.50, RMSEA = 0.052, CFI = 0.936, NFI = 0.913).

**TABLE 4:** Results for hypotheses testing (H1 and H2).

Path	Estimates	SE	CR	*p-value*	LLCI	ULCI	Decision
ML-CSR → EC	(β1) 0.39**	0.054	7.22	0.003	0.193	0.464	Accepted
WE → EC	(β2) 0.30**	0.044	6.82	0.000	0.128	0.391	Accepted

ULCI, upper-limit confidence interval; LLCI, lower-limit confidence interval; and **, significant values.

After confirming the direct effects for H1 and H2, the authors continued with the SEM for the second stage. This time, the authors introduced WE as a mediator in the structural model and checked the outcomes. The authors used bootstrapping option in AMOS to validate the mediating effect of WE. The bootstrapping method is more preferred to test the mediation effect as compared to Baron and Kenny (1986) conventional approach, which is criticized by different scholars including Hayes (2009), Zhao et al. (2010). The authors selected a large bootstrapping sample of 2000 for this purpose. In this regard, the empirical findings of the mediated model produced significant results thus providing support in the favor of Hypothesis 3 (H3). Therefore, it is statistically supported that WE is a partial mediator between ML-CSR and EC (β3 = 0.16, *p* < 0.05). It is to be observed that beta value is reduced (β1 = 0.39 to β3 = 0.16) but still remained significant, which is an evidence of the partial mediation. Thus, all three hypotheses (H1, H2, and H3) of this analysis were significant. Likewise, the model fit values of mediated model were also significant, in fact, these values were even more better this time as compared to the direct effect model, thus suggesting a further confirmation that there is more fit to the data (χ^2^ = 2336.620, *df* = 988, *p* < 0.01 χ^2^/*df* = 2.365, RMSEA = 0.041, CFI = 0.944, NFI = 0.927). These results of the mediated model have been reported in Table 5.

**TABLE 5:** Mediation results for H3.

Path	Estimates	SE	*Z-*Score	*p-value*	LLCI	ULCI	*R* ^2^	Decision
ML-CSR → WE → EC	(β3) 0.16**	0.022	7.27	0.000	0.102	0.196	0.37	Accepted

ULCI, upper-limit confidence interval; LLCI, lower-limit confidence interval; and **, significant values; S.E, standard error.

### Reverse Causation

**FIGURE 1:**
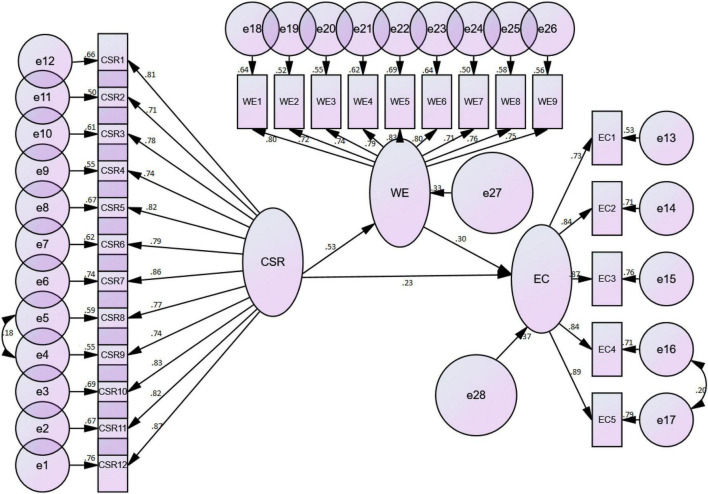
Presents the structural model of the hypothesized relations.

Lastly, the authors also tested reverse causality due to the possibility that the engaged workers may have a favorable perception about their hotel (for example, it is likely that they may have unusual positive feelings about their hotel’s CSR activities). Therefore, checking for reverse causality was logical here (Katz, 2006). To do this, the authors analyzed the same proposed structural model (Figure 1) but with reverse directions. Although the model fit values were similar to the baseline model, the results of mediation (reverse causation from EC to ML-CSR through WE as a mediator) were not significant this time (β = 0.013, *p* = 0.93). The absence of reverse causation was confirmed in the current analysis.

## Discussion

This analysis validates all three proposed hypotheses (H1, H2, and H3) framed with the help of theoretical support and related literature in previous sections. Furthermore, ML-CSR activities of a hotel are confirmed in this study to be helpful in boosting EC in a positive way. Similarly, the importance of the underlying mechanism of how this relationship is explained through the mediating effect of WE was also highlighted. The argument here is that CSR activities of a hotel are likely to create two kinds of reciprocity effects, restricted reciprocity, and generalized reciprocity. In so far as restricted reciprocity is concerned, the ML-CSR activities of a hotel create positive feelings among the employees, and they feel obliged in response to such CSR activities that are related to the employees. Generalized reciprocity is concerned with the general feelings of the employees about their hotel when a hotel is involved in general CSR-related activities not specifically related to employees. These general CSR activities are also well taken by the employees as member of a macro group of stakeholders including, society, and the environment. In fact, Gürlek and Tuna (2019) also acknowledged this effect of general reciprocity of CSR activities in the context of the hotel sector of Turkey. In like manner, Farooq et al. (2014) also mentioned restricted and generalized reciprocity as an outcome of CSR. Thus, when hotels are engaged in ML-CSR activities, employees are likely to repay such organizations with their greater level of WE and creativity.

More specifically, the current analysis extends the work of Glavas and Piderit (2009), who acknowledged that employees’ CSR perception is directly related to their creativity but failed to observe the underlying mechanism of WE as a mediator. The current analysis indicates that despite the direct effect of ML-CSR on creativity, CSR perception of the hotel employees augments their creativity through WE as a mediator. Moreover, this analysis also extends the work of Gürlek and Tuna (2019), who acknowledged the positive link of ML-CSR activities with WE, but they did not consider that this relationship further can induce EC, so imperative where competition is the sine qua non for every sector. Different extant researchers have also reported the direct and indirect effect of CSR on EC in different cultures and industries (Brammer et al., 2015; Abdelmotaleb et al., 2018; Hur et al., 2018). The current work also extends the boundary for the work of Nazir and Islam (2020a), who identified the relationship between ML-CSR and WE in the hospitality sector. However, they provided no further link on how better-engaged employees can be creative workers in response to ML-CSR. Lastly, this analysis extends the work of previous researchers in the same field (Chaudhary and Akhouri, 2018; Ko and Choi, 2020).

### Theoretical Implications

The current analysis augments the existing literature in many ways. For instance, the current analysis is a pioneering one in the hotel sector of Pakistan, taking into account the micro foundation of CSR to foster the extra role of employees such as their creative capability at the workplace. The previous studies in the domain of CSR were largely conducted in the domain of macro-CSR (Yang and Baasandorj, 2017; Cho et al., 2019; Franco et al., 2020). Moreover, the current work contributes to the existing literature of organizational psychology through the intervention of ML-CSR as an enabler to better involve employees in creative tasks through WE. Although previous studies tested the direct effect of ML-CSR on employees’ outcomes, the inclusion of WE in the current work provides another important contribution to the existing literature. Another important contribution of the current work is that it highlights the importance of micro-CSR in the field because, in previous studies, the CSR effect on employees was largely aggregated to the macro-level, wherein it was not possible to see the impact of CSR specific activities on employees. Perhaps this is the reason that most of the macro-level studies produced mixed results. The current analysis addresses this problem and helps to understand the underlying psychological process that boosts EC in response to CSR activities.

### Practical Implications

Besides theoretical implications, the current work has some important implications for practice. First, this work attempts to improve the understanding of the policymakers, more specifically, from the hotel sector of Pakistan to realize CSR as an enabler for EC. It is important to mention here that the current state of CSR affairs in most of the hotels is philanthropic. Serena Hotels can be put as a case in point here which initiated a CSR-related project with the name of “Karighar.” The aim of “Karighar” was to empower the community through technical and vocational training. Although since its establishment, the project has produced significant outcomes, the problem lies with its philanthropic orientation toward CSR. To explain further, from a philanthropic perspective, most of the hotels consider CSR for charity and donation purposes. Mövenpick Hotel Karachi may be put as another example that practices philanthropic CSR under the title of the “Kilo of Kindness” campaign. The policymakers should realize that philanthropic orientation is just one facet of CSR, and it does not imply that if an organization is following CSR to the extent of philanthropy, it is likely to reap the full benefits of such efforts (Ahmad et al., 2021c; Mahmood et al., 2021). In sum, this sector must go beyond the philanthropic orientation of CSR and consider it to create a solid base of competitive advantage by addressing CSR toward employees. Yet another important implication of the current study is that it argues that well-planned ML-CSR activities of a hotel can create an emotional pull among employees to stay committed to a socially responsible hotel for a long time. This point is very important for a sector that is globally characterized by a high turnover rate. Thus, using the ML-CSR strategy, a hotel is likely to reduce not only its turnover but also involve the employees in participating in extra roles to stimulate their group performance (the organization). Similarly, the findings of the current work are helpful for managers and policymakers to realize that they can better survive in the face of competition through EC as an outcome of CSR (Guo et al., 2021). In this regard, the employees can serve as a source to provide meaningful creativity for a hotel.

### Limitations and Directions for Future

The current work encountered certain limitations, which can open new avenues for future researchers. The first limitation of the current analysis is that it only considered upscale hotels in the country. Thus, for a better representation of this sector, future researchers should consider other hotels in their sample. Another limitation of the current analysis lies with its geographical concentration as the study considered only three large cities. Therefore, future researchers are suggested to consider other cities in their sample. For instance, they may include, Multan, Faisalabad, Sialkot, and Rawalpindi, etc. Yet another limitation is that the current work collected the cross-sectional data, which limits the causality of the variables. Hence future researchers are suggested to address this issue by considering longitudinal data design. Likewise, like in other regions of the world, the hotel sector of Pakistan was badly affected by the Covid-19 pandemic. During the pandemic era, due to the strict lockdown situation, many hotels remained empty as there were no visitors. For better generalizability, future CSR studies may consider this pandemic factor with respect to the hotel sector. Lastly the current research did not include any control variable (age, gender, etc.), therefore, it is suggested to consider such control variables in future studies.

## Conclusion

To conclude, the current research opens a new arena for the hotel sector of Pakistan as it attempts to introduce CSR as an enabler for workplace creativity, which is very important for this sector to remain competitive in the industry. The empirical findings of the current analysis revealed that employees’ CSR perception of their hotel serves as a base for the employees to identify with a hotel and put extra efforts to induce the overall performance of their hotel. Further, it is also revealed that CSR should not be considered a philanthropic motivator, it should be incorporated at further levels if a hotel wants to enjoy its full benefits, including EC. In this regard, it is recommended that the policymakers engage their employees in designing different CSR-related strategies so that they can better develop these strategies to produce better outcomes not only for the employees but also for society and the environment. Lastly, policymakers need to provide their employees with a better workplace characterized by CSR orientation if they want to use their employees as a source of creativity to keep their business alive in this sector.

## Data Availability Statement

The raw data supporting the conclusions of this article will be made available by the authors, without undue reservation.

## Author Contributions

All authors contributed to conceptualization, formal analysis, investigation, methodology, and writing and editing the original draft.

## Conflict of Interest

The authors declare that the research was conducted in the absence of any commercial or financial relationships that could be construed as a potential conflict of interest.

## Publisher’s Note

All claims expressed in this article are solely those of the authors and do not necessarily represent those of their affiliated organizations, or those of the publisher, the editors and the reviewers. Any product that may be evaluated in this article, or claim that may be made by its manufacturer, is not guaranteed or endorsed by the publisher.
